# Echocardiographic Findings in Asymptomatic Mediastinal Lymphoma Survivors Years after Treatment Termination

**DOI:** 10.3390/jcm12103427

**Published:** 2023-05-12

**Authors:** Izabela Nabiałek-Trojanowska, Hanna Jankowska, Grzegorz Sławiński, Alicja Dąbrowska-Kugacka, Ewa Lewicka

**Affiliations:** 1 First Department of Cardiology, Faculty of Medicine, Medical University of Gdańsk, 80-210 Gdańsk, Poland; 2 Department of Cardiology and Electrotherapy, Faculty of Medicine, Medical University of Gdańsk, 80-210 Gdańsk, Poland; lek.grzegorzslawinski@gmail.com (G.S.);; 3 Division of Cardiac Diagnostics, Medical University of Gdańsk, 80-210 Gdańsk, Poland; hjankowska@gumed.edu.pl

**Keywords:** cardiotoxicity, radiation injuries, dobutamine stress echocardiography, cancer survivors, mediastinal lymphoma, cardiovascular diseases

## Abstract

Patients treated due to mediastinal lymphomas are at risk of cardiovascular complications, as they receive chemotherapy, usually containing anthracyclines, often combined with thoracic radiotherapy. The aim of this prospective study was to assess early asymptomatic cardiac dysfunction using resting and dobutamine stress echocardiography (DSE) at least 3 years after the end of mediastinal lymphoma treatment. Two groups of patients were compared: those treated with chemoradiotherapy and those exclusively treated with chemotherapy. Left ventricular contractile reserve (LVCR) during DSE was assessed using changes in LV ejection fraction (LVEF), LV global longitudinal strain (LV GLS), and a novel parameter—Force, which is the ratio of the systolic blood pressure to the LV end-systolic volume. The study included 60 patients examined at a median of 89 months after the end of treatment. Resting echocardiography showed normal LVEF of 58.9 ± 9.6%, borderline LV GLS of −17.7 ± 3%, decreased mean stroke volume (SV) of 51.4 ± 17 mL, and indexed SV of 27.3 ± 8 mL/m^2^, and the right ventricular free wall longitudinal strain (LS) was impaired in some patients but not in all. There were no significant differences between the groups, with the exception of arterial hypertension, which was more common in the chemotherapy group (32% vs. 62.5%, *p* = 0.04). In resting echocardiography, only LV posterior wall LS differed significantly and was impaired in patients treated with chemotherapy (−19.1 ± 3.1% vs. −16.5 ± 5.1%, *p* = 0.04). DSE, performed in 21 patients after a median of 166 months from the end of cancer treatment, detected new contractility disorders in 1 patient (4.8%) and decreased LVCR in the majority of patients when determined using changes in LVEF or LV GLS, and in all patients when assessed with changes in Force. Conclusions: Most asymptomatic mediastinal lymphoma survivors showed preserved ventricular function on resting echocardiography. However, all of them showed impaired LV contractile reserve on DSE, as assessed with a simple parameter—Force. This may indicate subtle LV dysfunction and confirms the need for long-term monitoring of patients with potentially cardiotoxic cancer treatment.

## 1. Introduction

Progress in anticancer therapies in recent decades has significantly improved disease prognosis [1,2]. However, despite a constant improvement in treatment methods, complications of some anticancer therapies may still substantially worsen quality of life and survival. It is emphasized in the current European Society of Cardiology (ESC) guidelines on cardio-oncology [3] that the risk of cardiac dysfunction depends on the total dose of doxorubicin used, irradiation technique and dose, and the patient’s clinical status. Patients at increased risk of cardiotoxicity should be monitored within 12 months after treatment with doxorubicin, when the risk of left ventricular (LV) dysfunction occurrence is the greatest. On the other hand, the risk of complications due to radiotherapy is life-long and increases the longer the time after its completion. The combination of chemotherapy and thoracic irradiation increases the cardiotoxicity of anticancer treatment [4]. Therefore, control of cardiovascular risk factors (CVRF) is of great importance in cancer patients to prevent cardiotoxicity from cancer treatment.

The present study aimed to evaluate cardiac function by means of echocardiography in asymptomatic patients who underwent chemoradiotherapy or chemotherapy alone for mediastinal lymphoma. Apart from standard visual assessment of global and regional systolic ventricular function, a speckle-tracking echocardiography (STE) was used to examine LV and right ventricular (RV) systolic function at rest and during dobutamine stress echocardiography (DSE). We also wanted to assess the usefulness of the new parameter, which is Force, in detecting subclinical LV dysfunction depending on the applied oncological treatment. Force is calculated as the ratio of systolic blood pressure to LV end-systolic volume. This novel variable is easy to obtain and does not increase the imaging time. Force reflects a decrease in the pressure-volume loop slope, a decrease in systolic blood pressure, and transient ischemic LV dilatation during DSE. All these results are associated with a poor prognosis and allow the sensitivity of DSE to be extended to LV dysfunction [4,5,6]. We aimed to compare the results of echocardiography at rest and during DSE in patients treated exclusively with chemotherapy or chemoradiotherapy, with the hypothesis that combined treatment is associated with a higher incidence of cardiac injury.

## 2. Materials and Methods

### 2.1. Patient Population

The study was prospective and included patients treated with chemoradiotherapy or chemotherapy alone for mediastinal lymphoma at least 3 years prior to enrollment. Data on their treatment were obtained from local oncology centers: the Department of Haematology and Transplantology, and the Department of Pediatrics, Hematology and Oncology of the Medical University of Gdansk and the Provincial Cancer Center in Gdansk, Poland. The exclusion criteria were heart disease diagnosed prior to study enrollment, current anti-cancer treatment, and lack of the patient’s consent to participate in the study. All patients were under the care of the Cardio-Oncology Outpatient Clinic, where great attention was paid to proper control of CVRF.

The study protocol included a medical history, physical examination, CVRF and comorbidities assessment, transthoracic echocardiography (TTE), DSE, and Charlson comorbidity index. Fasting blood samples were collected to determine blood counts and concentration of brain natriuretic peptide (BNP), creatinine, glucose, and parameters of the lipid profile (total cholesterol and its fractions). Dyslipidemia was defined as a low-density lipoprotein level (LDL cholesterol) > 130 mg/dL or statin use. Chronic kidney disease was recognized when the estimated glomerular filtration rate was <60 mL/min/1.73 m^2^.

Data on the treatment of lymphoma were analyzed, which included the chemotherapy regimen and the radiation technique, a total and fractional dose of radiation therapy, and the time since completion of anti-cancer therapy. The control group consisted of 11 healthy individuals, matched for age and sex, who were referred for a DSE examination for the diagnosis of coronary artery disease (CAD). The study was approved by the local Institutional Ethics Committee (NKBBN/419/2017) and written informed consent was obtained from all patients.

### 2.2. Rest Echocardiography

TTE was performed using a Vivid S6 or Vivid E95 cardiovascular ultrasound system (GE Healthcare, Little Chalfont, UK), and images were analyzed offline using a GE EchoPAC PC work station (GE, Boston, MA, USA). All measurements were made using two-dimensional (2D) imaging and speckle-tracking echocardiography, and were performed according to the recommendations of the American Society of Echocardiography (ASE) [5]. Left ventricular systolic function was determined on the basis of LV ejection fraction (LVEF), stroke volume (SV) and stroke volume index (SVi), and LV global (LV GLS) and segmental longitudinal strain (LS). The LVEF was calculated using the biplane Simpson method, and LVEF < 54% in women and <52% in men was considered abnormal. The SV was calculated as a difference between the LV end-diastolic and end-systolic volumes, and values < 60 mL were considered abnormal. The SV_i_ indicated SV depending on the patient’s body surface area, and values < 35 mL/m^2^ were considered abnormal [5,6,7,8,9].

Right ventricular systolic function was determined by assessing the RV fractional area change (FAC), tricuspid annular plane systolic excursion (TAPSE), RV peak systolic velocity (RV s’) obtained from pulsed-wave tissue Doppler imaging, and RV free wall LS. The LS cut-off values were adapted from the ASE guidelines: LV LS below −16% (absolute value) was considered abnormal, LS between −18% and −16% borderline, and RV free wall LS below −20% (absolute value) abnormal [5,6,7,8,9].

### 2.3. Dobutamine Stress Echocardiography

DSE was performed in addition to routine echocardiography, according to a standard protocol with a graded dobutamine infusion starting from a dose of 5 µg/kg per minute. It was increased every 3 min to a dose of 10, 20 (low dose), 30, and finally 40 µg/kg per minute (peak dose). The goal of dobutamine infusion was to achieve 85% of the maximal predicted heart rate for the patient’s age. In patients without a planned increase in heart rate, atropine 0.5 mg to 1 mg was administered.

During DSE, images were obtained in standard views: in the parasternal long axis; parasternal short axis (at the mitral valve, midventricular, and apical level); apical 4-, 2-, 3-chamber views; and the RV-focused view. All measurements were performed at rest, at low and peak doses of dobutamine, and in the recovery with a frame rate of at least 50 per second. The test was terminated at the targeted heart rate, infusion protocol completion, in cases of systolic blood pressure (BP) increase over 220 mmHg or diastolic BP rise to over 120 mmHg, when symptomatic hypotension or a drop in systolic BP of more than 40 mmHg occurred, or in cases of symptomatic ischemia or serious arrhythmia.

To evaluate changes in LV contractility during DSE, LV contractile reserve (LVCR) was determined. Preserved LVCR was defined as an increase in LVEF or LV GLS of at least 20% when compared with baseline values [10,11]. The LVCR was also assessed using a novel parameter: left ventricular elastance reserve (Force), defined as the ratio of non-invasive systolic BP to LV end-systolic volume. Contractility reserve was estimated from the quotient of the Force measured at peak dobutamine dose to the baseline Force. The LVCR was considered normal when it was >2.0 [12,13,14]. Flow reserve was defined as an increase in SV of at least 20% from the baseline [15]. The occurrence of ischemia during DSE was indicated by the detection of new contractility abnormalities during visual assessment in at least 2 segments of the LV [16].

In order to reduce measurement errors of echocardiographic parameters, patients with poor acoustic windows were excluded from analysis.

### 2.4. Statistical Analysis

All data are presented as mean ± standard deviation or number and percentage. A normal distribution was confirmed via Shapiro–Wilk test and further analyses were made using Student’s *t*-test and Pearson’s correlation. When analyzing variables without normal distribution, the Mann–Whitney U test, and ANOVA, a Spearman’s rank correlation coefficient were used.

The patients were divided into two groups according to the applied lymphoma treatment: chemoradiotherapy or exclusively chemotherapy. The results obtained in both groups in echocardiography at rest and during DSE were compared. The DSE results were compared between the study patients and the control group. All calculations were performed using Statistica software, version 13, Dell Inc. 2016, Tulsa, OK, USA. The *p* value < 0.05 was considered significant.

## 3. Results

The study group consisted of 60 patients, 36 (60%) women and 24 (40%) men with a mean age of 49 ± 15 years (range 18–79), who underwent treatment between 1976 and 2018. Demographic and clinical data of the study group are presented in Table 1.

The majority of patients were treated for Hodgkin lymphoma (60%) or diffuse large B-cell lymphoma (32%), while 8% were treated for other types of non-Hodgkin lymphoma. All patients received systemic chemotherapy, most commonly according to the R-CHOP (rituximab, cyclophosphamide, doxorubicin, vincristine, prednisone; 42% of patients) or ABVD regimen (doxorubicin, bleomycin, vinblastine, dacarbazine; 42% of patients). The mean total doxorubicin dose in the study group was 292 ± 87 mg/m^2^ (250–399 mg/m^2^). There were no significant differences in chemotherapy regimens between the groups. Additionally, in 28 (46.7%) patients, irradiation of mediastinal lymph nodes was performed with a mean dose of 34.3 ± 8.8 Gy and in a mean of 18.7 ± 6 fractions.

With regard to radiation therapy, in eight patients who were treated in the years 1976–2008, data on the technique of irradiation were missing. Among the remaining 20 patients, for 12 patients a new radiotherapy technique, intensity-modulated radiation therapy (IMRT), was used, and shielding was used with 3 patients.

When assessing CVRF, arterial hypertension and dyslipidemia were most commonly recognized and arterial hypertension was more common in patients treated exclusively with chemotherapy. Additionally, the Charlson comorbidity index was greater in these patients.

Echocardiography was performed at a median of 89 (38–508) months after completion of anticancer treatment, and results are presented in Table 2. The dimensions of the heart chambers and LVEF were in normal ranges and without significant differences between groups. The mean LV GLS was borderline (−17.7 ± 3.2%), with no difference between the groups. In 12 patients (20%), LVEF was decreased, while abnormal LV GLS was found in 14 (23.3%) and borderline in 15 (25%) patients. Detailed analysis of the LV segmental LS revealed abnormal values in the anterior wall and in anterior and inferior parts of the interventricular septum. However, only LV posterior LS was significantly different between groups. The mean SV and SV_i_ were decreased, but did not differ between groups. RV systolic function reflected by TAPSE, RV FAC, RV s’ was not impaired. RV free wall LS was abnormal in 25 patients, which resulted in a decreased mean value in all patients (−19.9 ± 6.3%), although without significant differences between the groups.

Twenty-four patients were initially disqualified from DSE due to a poor acoustic window on echocardiography and seven patients did not attend DSE. In eight patients, DSE was terminated prematurely because of the occurrence of serious abnormalities, and they were excluded from further analysis. One patient had a premature termination of DSE due to frequent premature ventricular beats, three patients reported chest pain, three had a symptomatic BP drop, and one patient developed transient second degree atrioventricular block (type one). Thus, DSE results were analyzed in 21 (35%) patients with a mean age of 51 ± 13 years, after a median of 166 months from the completion of anticancer therapy. Demographics of these patients and results of DSE are presented in Table 3. Apart from dyslipidemia, there were no significant differences between the groups. In patients treated with chemoradiotherapy, the mean total radiation dose was 38.1 ± 12 Gy. There were no differences in the parameters assessing the LV systolic function, apart from LVEF, which was lower at baseline and at the peak dobutamine dose in patients treated exclusively with chemotherapy. The LVCR calculated using Force was decreased without significant differences between groups. In both groups, there was no increase in LVEF of 20% with the peak dose of dobutamine. There were no differences between groups in the parameters describing the RV systolic function. There were no ischemic changes on the ECG during DSE, but in one (4.8%) patient after chemotherapy the test was positive, as new regional LV contractility disorders occurred. In this patient, coronary angiogram showed significant stenosis of the right coronary artery, which was treated with angioplasty and the implantation of a drug eluting stent.

All subjects in the control group had a negative DSE result for the diagnosis of CAD. Table 4 presents data on clinical characteristics and echocardiographic parameters obtained in all patients studied in comparison with age- and sex-matched healthy controls. There were no significant differences in comorbidities apart from dyslipidemia, which was more common in the study group (*p* = 0.049). LVCR obtained from Force was lower in cancer survivors compared with controls (*p* = 0.01). There were no significant differences in other echocardiographic parameters.

Results of the DSE performed in one of the patients treated with chemotherapy are shown in Table 5. This was a 45-year-old man examined 14 years after chemotherapy (ABVD) for Hodgkin’s lymphoma. The total dose of doxorubicin used was 380 mg/m^2^. He reported no symptoms, cardiovascular risk factors, or comorbidities. Baseline BP was 134/83 mmHg, and heart rate was 62 bpm. Resting echocardiography showed LVEF 56%, LV GLS −18.5%, and no contractility disorders. DSE was performed with the peak dobutamine dose of 50 mcg/kg/min and the addition of 1 mg atropine.

## 4. Discussion

Using modern echocardiography techniques at rest in asymptomatic mediastinal lymphoma survivors years after the completion of cancer therapy, we found well-preserved LV and RV systolic function, with no differences between patients treated with chemoradiotherapy containing doxorubicin and chemotherapy alone. However, DSE revealed impaired LV contractile reserve in both groups. Our study is the first to use a new parameter—Force in cardio-oncology to assess LVCR. LVCR obtained from Force was impaired in all cancer survivors, with no differences between groups, indicating patients with early LV dysfunction. Force allowed early detection of subtle LV injury, while LVCR assessed using standard parameters, LVEF and LV GLS, was insufficient. According to Wierzbowska-Drabik et al., Force calculated at peak DSE stage was correlated with the extent of the coronary artery disease (CAD) assessed using SYNTAX and Gensini scores, while LVEF and LV GLS values showed a poor relationship. The authors explained this by hypercontraction of non-ischemic LV segments, masking regional impairment of contractility and improving LVEF and LV GLS values [14]. In addition, the usefulness of the LV GLS assessment is limited at high heart rates due to under-sampling, resulting in lower accuracy of the strain calculation. It is caused by speckle de-correlation, because a significant part of the movement takes place between successive frames [17,18]. The addition of Force takes into account the effects of changes in LV end-systolic pressure (ESP) and LV end-systolic volume (ESV) in response to exercise on LVCR. In a healthy heart, an increase in ESP and a decrease in ESV are expected, otherwise it is associated with a poor prognosis. The decrease in SBP and transient LV dilatation observed during DSE, with a known negative prognostic value, will also be reflected by the LVCR obtained from the Force. Force is a simple parameter to calculate; it does not require any dedicated software or additional imaging, and its addition does not significantly extend the time of interpretation of DSE results, but enriches the conclusions with a parameter correlating the changes of the afterload to the preload. Importantly, it does not require any special imaging techniques such as, e.g., tissue Doppler imaging for post-systolic stress analysis or dedicated software for myocardial work assessment and post systolic stress obtained from speckle-tracking echocardiography.

It has been shown that mediastinal lymphoma survivors have a four–six times higher cardiovascular risk compared with the general population [19], and LV systolic dysfunction may occur many years after the end of anticancer therapy [17]. The incidence of cardiac impairment depends on the treatment used. Anthracyclines with known cardiotoxic potential [18] were used in all patients included in the study, and the total dose was 292.0 ± 87 mg/m^2^. In addition, 28 patients, i.e., almost half of the study group, were treated with mediastinal irradiation, which increases the risk of cardiac damage [20].

According to the newest ESC guidelines [3], patients included in our study were at high CV toxicity risk. In patients treated with chemotherapy, it was because they all received ≥250 mg/m^2^ of doxorubicin (250–399 mg/m^2^). In patients who underwent chemoradiotherapy, the risk was even very high, as the prescribed irradiation dose was 34.3 ± 8.8 Gy and the cumulative doxorubicin dose was ≥100 mg/m^2^.

The results obtained in this study were not in line with our expectations. Despite the high CV toxicity risk, all patients were asymptomatic, echocardiography at rest was normal, and DSE revealed CAD in only one patient. Additionally, the DSE results did not differ between the groups. However, the risk of radiation-induced cardiotoxicity is time-dependent: the greater the longer the time elapsed since this treatment. In our group, it was (median) 127 months, and perhaps it was still too short to cause LV systolic dysfunction in resting echocardiography. Heidenreich et al. diagnosed CAD via stress test in 14% of patients after a median of 15 years from mediastinal irradiation with a dose of at least 35 Gy [21]. In our group, DSE revealed new contractility disorders only in 1 out of 21 patients.

It is known that restrictive control and management of CVRF significantly reduces the risk of cardiac dysfunction occurrence [22,23,24]. All patients from our study were at least annually reviewed for CVRF and educated to promote a healthy lifestyle. That has allowed for strict control and management of CVRF, which probably reduced the risk of cardiac dysfunction. Moreover, in 54% of patients treated with chemoradiotherapy, modern techniques of radiotherapy (IMRT) and shielding were used, which are associated with lower cardiotoxicity potential. However, in the case of patients treated exclusively with chemotherapy, dyslipidemia was found more often, which may explain why there were no significant differences in DSE results. However, we approach the results of DSE with some reserve, as we were able to perform this examination only in one-third of patients. We decided to perform DSE instead of exercise stress echocardiography to ensure the good imaging quality that is required for adequate GLS analysis. Unsatisfactory cardiac imaging is often found in patients after chest radiotherapy and in the case of obesity [13,25,26,27], which was common in the study group (27%). These factors may limit the use of DSE in long-term monitoring after chest irradiation, and in these patients coronary computed tomography angiography (CCTA) should be performed.

When assessing the location of longitudinal deformation disorders at rest, we found only a significantly lower posterior wall LS in patients treated with chemotherapy. This is in contrast to the literature data, as it is the RV, interventricular septum, and LV anterior wall that are more at risk of injury because this region receives the highest doses of irradiation during mediastinal radiotherapy. What is more, similar localization of myocardial injury detected by LV LS was described by some authors in patients treated with anthracycline chemotherapy alone [27,28,29,30].

In our study, we assessed LVCR during DSE using three methods: calculating the LVEF and LV GLS increase and Force. Force is the proportion of systolic blood pressure to LV end-systolic volume. It mirrors LV elastance and correlates the changes of the afterload to the preload [13]. The Force-based LVCR was validated according to the type of stress test used and had different reference values. With DSE or exercise testing, the LVCR is maintained if it is >2.0, and >1.1 with dipyridamole or adenosine infusion because their inotropic effect is weaker [13]. The LVCR was impaired in the majority of our patients, regardless of how it was assessed. However, both the LVCR assessed with changes in LVEF and LV GLS indicated a significantly lower CV risk compared with LVCR evaluated using Force. LVCR assessed with Force was reduced in all studied patients and preserved in 82% of healthy subjects. This parameter may indicate concealed LV dysfunction among asymptomatic cancer survivors with normal LVEF and LV GLS at rest. This finding may suggest possible susceptibility to further LV deterioration, especially in hypertensive patients or in the presence of other CVRF. Therefore, ongoing monitoring for LV deterioration is mandatory, as is the need to carefully manage CVRF. Nevertheless, due to the small number of patients undergoing DSE, these observations require confirmation in a larger group of patients and with further follow-up to determine whether impaired LV elastance, reflected by Force, correlates with subsequent coronary events.

### Limitations of the Study

Patients were treated with radiotherapy over many years and, thus, using different radiotherapy techniques, which could have influenced the results we obtained. In patients treated outside the Medical University of Gdansk, we did not obtain data on radiotherapy treatment, which did not allow us to establish the relationship between irradiation parameters and echocardiographic results. The size of the groups of patients in whom the DSE study was performed was small; therefore, we look critically at the obtained results and we realize that it still needs to be confirmed in larger groups of patients. Three-dimensional echocardiography, which would allow for a more accurate assessment of ventricular systolic function, including right ventricular ejection fraction (RVEF), was not performed. A comparison of Force with other methods of LV global systolic function assessment, such as dP/Dt, myocardial work, or post-systolic stress, is lacking.

## 5. Conclusions

Most asymptomatic mediastinal lymphoma survivors years after treatment with chemoradiotherapy or chemotherapy showed normal ventricular function on resting echocardiography. However, all of them had impaired LV contractile reserve on DSE, as assessed with a novel parameter—Force. The usefulness of Force assessment in cardio-oncology is promising and may indicate patients at risk of future LV dysfunction in whom conventional screening at rest does not yet reveal LV impairment. Therefore, long-term monitoring of patients after potentially cardiotoxic oncological treatment is necessary, as well as close control of cardiovascular risk factors in this group of patients.

## Figures and Tables

**Table 1:** Demographic and clinical characteristics of the study group at the time of echocardiographic examination and depending on the lymphoma treatment.

Parameter	All Patients *n* = 60	Chemoradiotherapy Group *n* = 28	Chemotherapy Group *n* = 32	*p*
Age, years	49 ± 15 (18–79)	46 ± 16 (18–79)	52 ± 15 (20–75)	0.12
Males, *n* (%)	24 (40)	9 (32)	15 (47)	0.33
Cardiovascular risk factors:				
arterial hypertension, *n* (%)	29 (48)	9 (32)	20 (62.5)	**0.04**
dyslipidemia, *n* (%)	26 (43)	10 (35.7)	16 (50)	0.36
diabetes, *n* (%)	6 (10)	1 (3.6)	5 (15.6)	0.43
current/former smoker, *n* (%)	15 (25)	4 (14.2)	11 (34.4)	0.18
obesity, (BMI > 30 kg/m^2^), *n* (%)	16 (27)	6 (21)	10 (31)	0.81
chronic kidney disease, *n* (%)	8 (13.3)	2 (7.2)	6 (18.8)	0.45
Charlson comorbidity index, median (quartile 25%, quartile 75%)	0.0 (0.0; 2.0)	0.0 (0.0; 1.0)	1.0 (1.0; 2.0)	**0.027**
Laboratory parameters:				
BNP, pg/mL	49.8	49.6	50.0	0.80
Cumulative doxorubicin dose, mg/m^2^	292.0 ± 87	284.1 ± 101	298.9 ± 73	0.51
Median time from cancer treatment termination, months	89	127	85	0.08

BMI—body mass index, BNP—brain natriuretic peptide.

**Table 2:** Results of the echocardiographic examination according to the applied lymphoma treatment.

Parameter	Chemoradiotherapy Group *n* = 28	Chemotherapy Group *n* = 32	*p*
LVEF, %	60.4 ± 9.3	57.5 ± 9.9	0.61
patients with decreased LVEF, *n* (%)	4 (14.3)	8 (25.0)	0.48
LV GLS,%	−18.0 ± 2.9	−17.4 ± 3.5	0.51
patients with an abnormal LV GLS, *n* (%)	6 (21.4)	8 (25.0)	0.82
patients with a borderline LV GLS, *n* (%)	5 (17.9)	10 (31.3)	0.38
inferior part of interventricular septum LS, %	−16.1 ± 3.9	−16.1 ± 3.7	0.99
anterior part of interventricular septum LS, %	−15.9 ± 4.8	−16.5 ± 5.5	0.66
anterior wall LS, %	−15.9 ± 4.1	−15.8 ± 5.2	0.95
lateral wall LS, %	−17.5 ± 4.9	−17.3 ± 3.8	0.66
posterior wall LS, %	−19.1 ± 3.1	−16.5 ± 5.1	**0.04**
inferior wall LS, %	−19.0 ± 3.5	−17.9 ± 4.5	0.33
SV, mL	50.8 ± 17.2	51.9 ± 17.1	0.86
SV_i_, mL/m^2^	27.7 ± 8.7	27.0 ± 7.8	0.79
TAPSE, mm	21.6 ± 3.9	21.7 ± 3.7	0.96
RV FAC, %	42.7 ± 7.7	44.4 ± 10.0	0.86
RV s’, cm/s	12.8 ± 3.0	12.2 ± 2.0	0.84
RV LS, %	−21.2 ± 6.5	−18.8 ± 5.9	0.16

FAC—RV fractional area change, LS—longitudinal strain, LV—left ventricular, LVEF—LV ejection fraction, LV GLS—LV global longitudinal strain, RV—right ventricular, RV LS—RV free wall longitudinal strain, RV s’—RV peak systolic velocity, SV—stroke volume, SVi—stroke volume index, TAPSE—tricuspid annular plane systolic excursion.

**Table 3:** Demographics, clinical data, and results of dobutamine stress echocardiography performed in patients treated with chemoradiotherapy and those treated exclusively with chemotherapy.

Parameter	After Chemoradiotherapy *n* = 11	After Chemotherapy *n* = 10	*p*
Age, years	48 ± 14 (29–79)	53 ± 12 (31–71)	0.41
Males, *n* (%)	2 (18)	6 (60)	0.11
Cardiovascular risk factors:			
arterial hypertension, *n* (%)	3 (27)	7 (70)	0.11
dyslipidemia, *n* (%)	2 (18)	7 (70)	**0.049**
diabetes, *n* (%)	1 (9)	1 (10)	1.00
current/former smoker, *n* (%)	2 (18)	2 (20)	0.97
obesity (BMI > 30 kg/m^2^), *n* (%)	1 (9)	2 (20)	0.70
chronic kidney disease, *n* (%)	0	3 (30)	0.26
Charlson comorbidity index, median (quartile 25%, quartile 75%)	0.0 (0.0; 1.0)	2.0 (0.0; 3.0)	0.25
Cumulative doxorubicin dose, mg/m^2^	258.2 ± 77	299.0 ± 72	0.25
Median time from cancer treatment termination, months	179	105	0.10
LVEF at baseline, %	65.9 ± 4.7	57.9 ± 7.9	**0.01**
Patients with decreased LVEF at rest, *n* (%)	0	3 (30)	0.26
LVEF at low dose, %	72.6 ± 5.7	68.4 ± 6.8	0.16
LVEF at peak dose, %	74.2 ± 5.8	68.7 ± 4.1	**0.046**
LV GLS at baseline, %	−19.5 ± 1.2	−18.0 ± 3.2	0.18
Patients with an abnormal LV GLS at rest, *n* (%)	0	2 (20)	0.46
Patients with a borderline LV GLS at rest, *n* (%)	1 (9)	3 (30)	0.44
LV GLS at low dose, %	−23.8 ± 2.1	−23.3 ± 2.7	0.67
LV GLS at peak dose, %	−22.5 ± 2.5	−22.9 ± 2.9	0.72
LVCR assessed with ΔLVEF at peak dose, %	12.6	18.6	0.27
Patients with reduced LVCR assessed with LVEF at peak dose, *n* (%)	9 (82)	4 (40)	0.11
LVCR assessed with ΔLV GLS at peak dose, %	15.9	27.8	0.26
Patients with reduced LVCR assessed with LV GLS at peak dose, *n* (%)	8 (73)	4 (40)	0.22
LVCR obtained from Force at peak dose	1.10	1.14	0.45
Patients with reduced LVCR assessed with Force at peak dose, *n* (%)	11 (100)	10 (100)	1.0

LV—left ventricular, LVEF—LV ejection fraction, LV GLS—LV global longitudinal strain, LVCR—LV contractile reserve, Force—the ratio of the systolic blood pressure to the LV end-systolic volume.

**Table 4:** Clinical characteristics and the results of dobutamine stress echocardiography (DSE) in cancer survivors and in subjects from the control group.

Parameter	Study Group *n* = 21	Control Group *n* = 11	*p*
Age, years	51 ± 13 (29–79)	49 ± 13 (33–76)	0.87
Males, *n* (%)	8 (38)	5 (45)	0.75
Cardiovascular risk factors:			
arterial hypertension, *n* (%)	10 (52)	3 (27)	0.11
dyslipidemia, *n* (%)	9 (48)	2 (18)	**0.049**
diabetes, *n* (%)	2 (9.5)	1 (9)	1.00
current/former smoker, *n* (%)	4 (19)	5 (45)	0.23
obesity (BMI >30 kg/m^2^), *n* (%)	3 (14)	2 (18)	0.87
chronic kidney disease, *n* (%)	3 (14)	0	0.26
LVEF at baseline, %	62.1 ± 7.5	63.6 ± 4.7	0.54
Patients with decreased LVEF at rest, *n* (%)	3 (14)	0	0.53
LVEF at low dose, %	70.8 ± 6.4	71.7 ± 6.1	0.72
LVEF at peak dose, %	72.1 ± 5.8	76.5 ± 5.8	0.06
LV GLS at baseline, %	−18.8 ± 2.4	−19.5 ± 1.7	0.38
Patients with an abnormal LV GLS at rest, *n* (%)	2 (9.5)	0	0.68
Patients with a borderline LV GLS at rest, *n* (%)	4 (19)	2 (18)	0.98
LV GLS at low dose, %	−23.6 ± 2.4	−23.0 ± 1.9	0.50
LV GLS at peak dose, %	−22.6 ± 2.6	−23.1 ± 1.8	0.61
LVCR assessed with ΔLVEF at peak dose, %	16.1	21.1	0.18
Patients with reduced LVCR assessed with LVEF at peak dose, *n* (%)	13 (62)	1 (9)	**0.01**
LVCR assessed with ΔLV GLS at peak dose, %	21.3	19.0	0.92
Patients with reduced LVCR assessed with LV GLS at peak dose, *n* (%)	12 (57)	1 (9)	**0.03**
LVCR obtained from Force at peak dose	1.12	2.04	**0.01**
Patients with reduced LVCR assessed with Force at peak dose, *n* (%)	21 (100)	2 (18)	**0.0002**

**Table 5:** Results of dobutamine stress echocardiography (DSE) performed in a 45-year-old male treated 14 years earlier for Hodgkin’s lymphoma with chemotherapy using doxorubicin.

Parameter	Baseline	Peak Dobutamine Dose	LVCR Estimated at Peak Dose
LVEF	56%	69%	23.2%
LV GLS	−18.5%	−22.6%	22.2%
Force	1.614 mmHg/mL	1.542 mmHg/mL	0.955

For abbreviations, see Table 3. Resting echocardiography showed no abnormalities. Normal increases in LVEF and LV GLS were observed during DSE. LVCR estimated from LVEF and LV GLS increased by at least 20.0% at peak dose. In contrast, the LVCR obtained from Force (<2) indicated left ventricular injury.

## Data Availability

The raw data supporting the conclusions of this article will be made available by the authors, without undue reservations.
